# Obstacle avoidance and smooth trajectory control: neural areas highlighted during improved locomotor performance

**DOI:** 10.3389/fnbeh.2013.00009

**Published:** 2013-02-18

**Authors:** Jac Billington, Richard M. Wilkie, John P. Wann

**Affiliations:** ^1^Institute of Psychological Sciences, Faculty of Medicine and Health, The University of LeedsLeeds, UK; ^2^Department of Psychology, Royal Holloway, University of London EghamSurrey, UK

**Keywords:** steering, locomotion, fMRI, performance

## Abstract

Visual control of locomotion typically involves both detection of current egomotion as well as anticipation of impending changes in trajectory. To determine if there are distinct neural systems involved in these aspects of steering control we used a slalom paradigm, which required participants to steer around objects in a computer simulated environment using a joystick. In some trials the whole slalom layout was visible (steering “preview” trials) so planning of the trajectory around future waypoints was possible, whereas in other trials the slalom course was only revealed one object at a time (steering “near” trials) so that future planning was restricted. In order to control for any differences in the motor requirements and visual properties between “preview” and “near” trials, we also interleaved control trials which replayed a participants' previous steering trials, with the task being to mimic the observed steering. Behavioral and fMRI results confirmed previous findings of superior parietal lobe (SPL) recruitment during steering trials, with a more extensive parietal and sensorimotor network during steering “preview” compared to steering “near” trials. Correlational analysis of fMRI data with respect to individual behavioral performance revealed that there was increased activation in the SPL in participants who exhibited smoother steering performance. These findings indicate that there is a role for the SPL in encoding path defining targets or obstacles during forward locomotion, which also provides a potential neural underpinning to explain improved steering performance on an individual basis.

## Introduction

A crucial element of survival for most animals is the ability to move through their environment successfully; steering toward objects of interest (e.g., food) and avoiding collisions with dangerous objects (e.g., a predator or concrete barrier). Such locomotor tasks require the integration of several informational variables available within the visual scene (Wilkie and Wann, 2002). Optical flow from the visual scene can be used by a human observer to determine their current heading direction (Warren and Hannon, 1988) and this information may be sufficient to maintain a straight locomotor trajectory. Executing skilled, smooth steering maneuvers through a series of waypoints, however, requires information about future targets/obstacles to be taken into account (Fajen and Warren, 2003; Wilkie et al., 2008). It may not be necessary for explicit “path planning” to occur due to inertia within the steering system (Wilkie et al., 2008), but even a seemingly simple task such a steering around one stationary object to approach another stationary object requires a neural system sophisticated enough to simultaneously consider several environmental cues and rapidly execute a series of finely timed motor commands. To date, little research has been devoted to exploring the neural correlates of locomotion through an environment containing targets and obstacles, and no research has considered if individual performance is reflected in specific cortical regions.

### Heading detection

Research in both non-human primates and humans has revealed a network of cortical regions which show preferences toward global optical flow components which are indicative of self motion and provide valuable information regarding current heading. MST, a sub-region of the human motion complex (MT+) located in the superior temporal cortex has been proven to show robust activation to visual cues which are compatible with self motion (global expansion and rotation patterns) in primates (Duffy and Wurtz, 1995; Page and Duffy, 2008) and humans (Dukelow et al., 2001; Wall et al., 2008). Two further regions, however, seem to have greater specificity with respect to encoding cues to self motion: the ventral intraparietal region (VIP) and the cingulate sulcus visual region (CSv) (Wall and Smith, 2008). A specific problem to be dealt with during egomotion is distinguishing between head-centered optic flow which arises as a result of self motion through the environment, and the contributions to flow that arise from retinal motion, i.e., eye and head rotations. Not only does VIP respond to cues which are compatible with self motion (Schaafsma and Duysens, 1996) but the representation of heading in this area seems to be in head-centered coordinates suggesting that this region may play a role in canceling retinal motion which arises from eye movements when making heading calculations (Schaafsma and Duysens, 1996; Zhang et al., 2004; Zhang and Britten, 2011). Both CSv and VIP exhibit strong BOLD responses to single optic flow patches, but these responses diminish when these flow patches are surrounded by further flow patches (i.e., visual cues which are inconsistent with self motion); MST on the other hand only shows a marginal preference for single vs. multi patch flow stimuli (Wall and Smith, 2008). These findings suggest a more specific role for heading detection in VIP and CSv compared to MST.

Several studies have examined human cortical involvement whilst making judgments of heading. The earliest of these, by Peuskens et al. (2001) found that judging heading in response to optic flow stimuli caused increased activation in MT+ and a dorsal region of the right intraparietal sulcus (IPS). MT+ activation was attributed to featural and visuospatial attention to optic flow components, whilst parietal activation was thought to more specifically reflect the process of extracting heading estimates. The role of IPS in controlling locomotion has been observed in other studies, with the suggestion that this region is linked particularly with the representation of egocentric and body-centric coordinates (Maguire et al., 1998) with lesions to the parietal lobe leading to navigational impairments when retracing a journey shown from a egocentric viewpoint (Seubert et al., 2008).

### Tracking and steering

The evidence discussed so far highlights functional cortical regions associated with optic flow stimuli consistent with egomotion. Very few studies, however, have attempted to simulate visual-motor scenarios more akin to natural locomotor steering, where objects serve to delineate the desired future path. Field et al. (2007) found that the provision of path features on a moving ground plane (dynamic road edges) activated an area of the superior parietal lobe (SPL) which was not activated in the presence of optic flow from ground texture alone. A region anterior to this SPL area displayed heightened activation in response to steering errors, even when the errors were merely passively observed. During active steering, bilateral activation in the anterior cerebellum was observed, which Field et al. (2007) hypothesize may reflect the process of continually updating forward model predictions based on the sensory feedback about the consequences of motor commands. This exploration of locomotor steering was taken further by Billington et al. (2010), who implemented a behavioral paradigm used by Land and Horwood (1995) to look at the differential effects of having far-road cues (1.5 s ahead) as compared to immediate near-road boundaries. During a passive heading task Billington et al. (2010) found that a region of the SPL that extended into the medial IPS (mIPS) was activated when far road features were used to anticipate steering responses. This activation was not present when making the same heading judgments in the presence of either near road features or when facing opposite the direction of travel, neither of which provide prospective information.

The studies of Billington et al. (2010) and Field et al. (2007) suggest that the SPL is involved in encoding future path information, such as the location of targets and obstacles, which are indicative of impending changes in heading and using this for the purpose of accurately timing motor responses. Neurons in the parietal cortex have been shown to be specialized in such a way as to facilitate such functions. In primates the mIPS is thought to be part of the parietal reach region (PRR) which is activated during goal directed motor planning and execution (Cohen and Andersen, 2002), such as visually guided hand movements toward a target (Grefkes and Fink, 2005). Another crucial function of the SPL is its ability to show sustained activation during intended, but not executed, goal directed pointing (Fernandez-Ruiz et al., 2007). Fernandez-Ruiz et al. (2007) also found that the topography of activation was tied to the retinal image; thus activation in this area represents intended goals in visual coordinates. In general, then, the specializations of neurons in the SPL allow for effective and simultaneous tracking of current heading and encoding of object locations in egocentric space.

### Object locations in the visual scene

Locomotion through the environment is not always guided by a continuously demarked path. Alternatively, locomotion can involve continuously updating and predicting the future location of obstacles in relation to the self, and each other, in order to pursue a self-initiated pathway. A study by Wolbers et al. (2008) addressed the cortical basis of spatial updating of object location in the visual scene. An increasing BOLD response in the precuneus and dorsal precentral gyrus coincided with an increasing number of objects in the visual scene, with a crucial dissociation between the two areas: the precuneus showed an increase in response with object number irrespective of whether a verbal or pointing response regarding object location was required, whereas dorsal precentral activation only increased when pointing. This finding suggests that the precuneus may be crucial for integrating information regarding object location in space somewhat independently from intended actions. Research on both macaques and humans suggests that regions within the precuneus are strongly interconnected with the SPL (Parvizi et al., 2006; Margulies et al., 2009) and, furthermore, reciprocal connections potentially exist between the precuneus and areas such as MST and CSv (Leichnetz, 2001). Taken together these findings suggest a very general role for the precuneus in computing object location in space which may be used by cortical areas such as SPL, MST, and CSv that guide egomotion and support efficient navigation.

### The current study

This study aims to build upon our previous research regarding neural contributions to effective heading detection and effective steering along predetermined pathways (Field et al., 2007; Billington et al., 2010). As well as considering the neural basis of encoding future obstacles for the purpose of efficient locomotion, we will also consider whether individual differences in performance are manifested in terms of brain activation. The SPL has been found to be recruited when errors in road positioning are detected (Field et al., 2007) and encodes information regarding future path (Billington et al., 2010). The precuneus is associated with spatial updating independent of intended movements (Wolbers et al., 2008) and so may play a role in predicting the future location of objects in order to execute timely movements. Here we specifically aim to see if these regions are associated with smoother and more efficient steering performance in our participants. We presented participants with a series of slalom layouts that required them to either actively identify the appropriate pathway or passively track heading on a replay of one of their previous steering trials. We manipulated the extent to which participants were able to plan their slalom course in the steering trials by either presenting the entire course at the start of the trial, or by only presenting the cones sequentially as the trial preceded (giving participants ~3 s action response time to a cone). These manipulations firstly enabled us to identify regions which were additionally recruited in order to encode the locations of approaching objects for the purpose of efficient locomotion. Secondly, manipulating the presentation timing of object location allowed us to assess which brain regions responded to path planning (beyond the most immediate object). By recording joystick responses during these trials we were able to assess how individual performance varies with neural activation.

## Methods

### Participants

Fourteen neurotypical participants (10 female, 4 male) between 20 and 36 years of age (mean 28.31, *SD* = 4.51) took part in this study. All participants were right handed, with normal or corrected to normal eye sight. All participants were screened according to standard fMRI scanning guidelines and gave their consent to take part. This study was approved by a local ethical committee.

### Procedures

#### Stimuli presentation

Stimuli were presented to the participant via a NordicNeuroLab VisualSytem^©^ with integrated optical diopter correction (−5pt to +2pt). The OLED display had 30° horizontal × 23° vertical display (800 × 600 pixels), all of which was visible to the participants. This system also allowed the monitoring of eye movements during trials. Participants were asked to lie comfortably in the scanner and had the VisualSystem lowered onto their eyes. Interpupillary distance was measured in order to set the optimum goggle disparity and diopter correction was used on participants requiring corrective eyewear.

#### Slalom task

**Figure 1:**
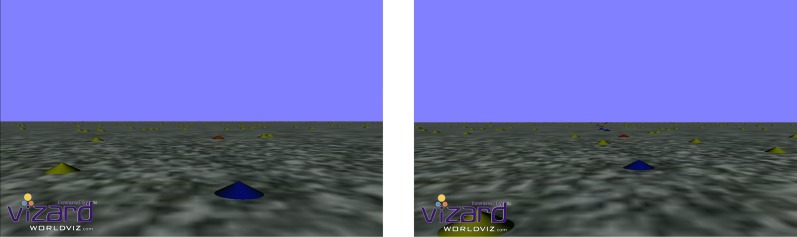
**Screen shots for both the Steer/Head Nr trials (left) and Steer/Head Pv trials (right).** Trials stimulus was interspersed with a screen the same color as the sky with a central cue indicating whether participants should respond to heading or actively steer.

Each condition was visually matched in that the lower half of the vertical axis contained a textured ground plane which provided optic flow cues as participants moved though the scene, and the upper half of the vertical axis contained a blue sky plane (see Figure 1). The participants' simulated viewpoint was set at 1.82 m above the ground and as such the nearest point of ground plane the participant could see was 4.8 m in front of them whilst the horizon was drawn ~190 m into the distance.

The general scene displayed for all conditions was of the ground plane strewn with numerous yellow cones placed at random locations (0.0079 cones/m^2^). This presented a cluttered scene with multiple object features but participants did not have to directly attend to these cones to complete the task.

Each trial block lasted 20 s with a between block rest duration of 7.9–8.5 s (random uniform distribution) in which a blue blank screen was presented. Each condition was presented 10 times in total over two separate runs. Five seconds before the start of each block a text cue appeared centrally on this screen instructing the participants as to the task that would appear in the following block.

If the text “Steer” was shown in the pre-cue period participants were presented with one of two conditions:

***Steer preview cones (SteerPv).*** Participants were presented with a scene described as above. In addition 13 red or blue cones were placed at intervals 15–640 m in front of the viewpoint, place laterally 1.2–4 m either side of an imaginary sum of sines pathway. Because of perspective projection only ~6 of these cones could be seen clearly at one time, with the remaining cones becoming clear as the participant moved toward the horizon. The amplitude of the underlying pathway was varied and the sign was reversed in 50% of trials to avoid the slalom path becoming predictable. Cone intervals were determined by placing cones at points of peak/trough amplitude in the imaginary pathway, resulting in cones being ~20 m apart on an average trial. Cones to the left of this pathway were red and cones to the right of this pathway were blue. Participants were moved forward at a constant speed of 8 m/s and instructed to steer right of the red cones and left of the blue cones in the smoothest manner possible. Participants travelled ~160 m during a trial, passing a total of 7–8 cones.

***Steer near cones (SteerNr).*** The SteerNr conditions presented the same steering task to the participants as in SteerPv but in this condition subsequent cones only became visible (fading in over a period of 0.5 s) to the participant when they were within ~25 m (3.13 s). Because of the projection characteristics (given an eye-height of 1.82 m and 40° vertical view angle) the ground was clipped from 4.8 m, which meant that the cone was only visible for 20.2 m (2.53 s). This condition therefore only allowed participants to see impending cones once they had already negotiated the preceding cone. Crucially, when they executed trajectories round a cone they could not take into account the position of the subsequent obstacle as you might if you were aiming to execute optimal steering commands.

If the text “Passive” was shown in the pre-cue period participants were presented with one of three conditions, which matched the visual content of the steering trials:

***Heading preview cones (HeadingPv).*** In the HeadingPv condition, participants did not have any active control of their movements with the joystick, but rather they had to indicate the angular velocity of their current heading whilst being steered through the same environment as in SteerPv. This instruction was conveyed to participants by explaining to them that they should respond as if they were a passenger in a car, mimicking the driver's actions. Heading could be detected from global optic flow patterns. In order to replicate the exact local/global motion and angular acceleration (aAcc) properties the trajectories were actual replays of one of the (randomly selected) previous three matched (i.e., near or preview) steering trials. This matching process is crucial as it allows us to use the heading trial as a baseline for the steering trials which is well matched in terms of local and global screen motion and the amount of motor movements made with the joystick. Comparing the two steering trials directly is likely to result in activations which merely reflect these confounding variables, as the nature of the near and preview steering trials dictates different steering strategies.

A series of cones were visible in this condition; however, they were randomly placed and not synced with the heading trajectory and therefore provided no indication about forthcoming changes in heading. This condition is a well matched control to SteerPv in terms of visual information and motor response; however, there are no requirements to plan steering responses using information regarding the spatial location of cones.

***Heading near cones (HeadingNr).*** HeadingNr followed the same principles as outlined for condition HeadingPv with the only difference being that the randomly placed cones faded in as the participants moved though the scene, yet still provided no indication as to impending changes in heading. This provided a well matched visual and motor control condition for SteerNr but required no need to attend to the proceeding cone.

***Baseline (BL).*** In the BL task the participants had to respond in the manner required for HeadingPv and HeadingNr to replays of previous steering trials. However, the scene contained no red and blue cones, only the yellow cones present in all conditions. This condition was included to provide a visual and motor baseline condition, without any trajectory planning or attentional distractions related to specific ground features.

#### Parietal eye field (PEF) localizer

We used a saccadic eye movement task to localize the parietal region thought to be the human homologue of the lateral intraparietal area in monkeys. A similar saccadic eye movement task was found to be an effective PEF localizer in our previous study and was used as an exclusive mask in order to remove cortical activation resulting from low level attentional effects and eye movements (Billington et al., 2010). We did not include an additional smooth pursuit localizer as it was thought unnecessary to subject participants to the additional scanning time required. In a previous unpublished pilot study we tested both pursuit vs. fixation and Saccade (Sacc) vs. Fixation (Fix) and found activations to be fairly equivalent, with a Sacc task resulting in slightly more extended activations. Also, in our previous study (Billington et al., 2010) activation in the cortical region associated with detecting future path showed no additional activation during Sacc vs. Fix, demonstrating that our Sacc localizer task did an efficient job of removing activations specific to eye movements. Thus, a Sacc task was deemed to be the most favorable in terms of providing a stringent exclusive mask to remove eye movement related cortical responses.

We presented participants with alternating Sacc and Fix blocks (16 s) and gave instructions to follow the dot on the screen at all times. The localizer lasted 256 s, with eight repetitions of each Sacc and Fix block. During Fix blocks the dot remained stationary in the center of the screen. During the Sacc block the dot position was randomly updated every 500 ms. The maximum horizontal eccentricity of the dot was 12.5° from the center of the screen and the maximum vertical eccentricity was 6.25° from the center of the screen.

### Behavioral data collection and analysis

Behavioral steering data was collected at 60 Hz using an MRI compatible joystick (MAG Concept, Redwood City, CA). This joystick was placed to the right hand side of the participant on the scanner bed so it was possible to steer comfortably with the joystick in the right hand for the duration of the scanning session (only right-handed participants were used). The maximum possible turning speed was 40.91°/s when the joystick was fully engaged to the left or right [participants used 14.18°/s (*SD* = 4.70) on average]. Participants were given the opportunity to practice steering outside the scanner with the same joystick until they felt confident about the device characteristics/sensitivity. Participants' continuous direction of heading (°) was calculated during each steering trial. To remove noise, heading values were subject to low pass filtering (25 Hz) using a Fast Fourier Transform operation. Average aAcc (°/s^2^) and angular jerk (°/s^3^) value were calculated for the SteerNr and SteerPv trials for each participant and used for both behavioral and fMRI analysis.

Eye tracking data was collected via an integrated NordicNeuroLab eye tracking camera (60 Hz) using Arrington software (Arrington Research, Scottsdale, AZ). Eye calibration grids were presented before both slalom runs and this data was used to standardize the data from each participant's slalom runs. This involved converting *x* and *y* pupil location values to screen coordinates in degrees from center (*h*: −15 to 15°, *v*: −11.5 to 11.5°) and filtering the small amount of data which fell out of this sample space. 10 out of the 14 participants provided clean eye tracking data for the left eye and for each of these participants the best run (least noisy) was selected for group analysis.

### fMRI data collection and analysis

#### Scanning acquisition and preprocessing

fMRI data were collected using a Siemens Trio 3 Tesla scanner with an eight-channel head array coil. Functional images were collected using 38 slices covering the whole brain (slice thickness 3 mm, interslice distance 0 mm, in-plane resolution 3 × 3 mm) with an echo planar imaging sequence (*TR* = 3 s, *TE* = 35 ms, flip angle =90°). All experiments in this study employed a block design and all fMRI data analysis was carried out using BrainVoyager software (Goebel et al., 2006). Prior to analysis, all images were corrected for slice timing using cubic spline interpolation. High pass (GLM-Fourier) temporal filtering was used to remove low frequency non-linear drifts in the data. Images were realigned to the first image in the first session. Finally, all images were smoothed with a full width half maximum Gaussian kernel of 4 mm. A high quality T1 weighted structural image (MDEFT) (Deichmann et al., 2004) was collected in each session, each fMRI run was co-registered to this structural image and then underwent a Talariach transformation so that each participants' data set was in a common space for group comparison.

#### First and second level analysis

Individual statistical contrasts were set up using the general linear model to fit each voxel with a combination of functions derived by convolving the standard haemodynamic response with the block design time series. Six additional regressors were added to each model in order to model potentially confounding rotational and translational minor head movements in *x*, *y* and *z* coordinates. Furthermore, the main experiment had an additional session regressor added to the model to account for acquisition of two separate data runs. Whole brain contrasts were carried out at a height threshold of *p* < 0.05 (FDR corrected) unless otherwise stated. ANCOVA analysis, for determining neural correlates of performance, was carried out at a height threshold of *p* < 0.001 [uncorrected (unc.)].

## Results

### Behavioral results

To steer a slalom participants must continually modify locomotor heading (°) giving rise to a change in angular velocity (°/s). Steering a smooth sinusoidal path would result in changes in aAcc (°/s^2^), whereas abrupt changes to the trajectory would result in increased angular jerk (°/s^3^). Mean values for both aAcc (aAcc) and angular jerk (aJrk) are shown in Table 1. Paired sample *t*-tests revealed that, on average, heading was changed at a larger accelerating rate for the SteerNr vs. SteerPv condition (aAcc mean diff. = 0.042, *t* = 2.883, *df* = 13, *p* < 0.05). However, there was no significant difference in the magnitude of angular jerk movements (aJrk mean diff. = 0.086) for the SteerNr vs. SteerPv trials. This would suggest that whilst participants are making more last minute rapid changes in heading for the SteerNr trial, this is not reflected in a large change in smoothness of steering.

**Table 1:** **Mean and standard deviation of aAcc (°/s^2^), AJrk (°/s^3^) and heading Lag (s)**.

**Condition**	**aAcc mean**	**aAcc *SD***	**aJrk mean**	**aJrk *SD***	**Mean lag, corresponding *r* values**	***SD* lag**
HeadingNr	4.554	2.182	227.707	123.555	0.889 (*r* = 0.375)	0.470
HeadingPv	4.504	2.429	217.993	99.297	0.888 (*r* = 0.374)	0.500
SteerNr	4.718	2.407	233.04	126.888		
SteerPv	4.467	2.316	233.0794	146.227		
BL	4.630	2.118	231.832	118.097	0.916 (*r* = 0.395)	0.480

A significant difference in aAcc between the SteerNr and SteerPv trials was noted (p < 0.05); no other significant differences were found.

No differences in aAcc and aJrk measures were found between the heading and steering trials. This is consistent with the fact that heading trials were replays of previous steering trials and therefore would elicit similar amplitude joystick movements from participants. Heading trials were associated with ~0.9 s lag in joystick response to on screen heading (see Table 1). There was no significant difference in heading lag values between passive replay trials (HeadingNr, HeadingPv, and BL) suggesting that the blue and red cones which were uninformative for the heading task did not have a distracting effect that potentially could have make the task more difficult than the BL task. Thus, these tasks provided a good visual and motor baseline to the steering task, without eliciting any additional cognitive demands due to a requirement to ignore the color coding of the cones.

### fMRI results

In order to ascertain whether there was any cortical activation present as a result of just seeing red and blue cones either continuously on the screen, or fading in as the trial progressed we compared both passive conditions, HeadingNr and HeadingPv, to the BL condition. Activation was present in parietal regions (bilateral precuneus; see Table 2), but only at a more lenient threshold (*p* < 0.001 unc.). This activation could reflect neural activation as a consequence of low level attentional effects or eye movements (which were not of interest to us). Thus, we employed the Sacc > Fix contrast from the independent PEF localizer as an exclusive mask on all subsequent whole brain contrasts in order to ensure our contrasts of interest were not including activation related to eye movement. Activation for this contrast can be seen in Figure 4 (shown in white) and all future reported experimental results are additional to this masked area.

**Table 2:** **Regions activated during whole brain contrasts, *x, y, z* coordinates are given in talairach space**.

**Contrast**	**Loci of peak voxel**	***x***	***y***	***z***	***N* voxels**	***t***	***p***
HeadingNr > BL	Precuneus	−23	−67	30	198	4.0	<0.001 (unc.)
		−4.9	−55	49	577		
		6.4	−53	45	441		
HeadingPv > BL	Precuneus	−21	−64	52	1179	4.0	<0.001 (unc.)
		5.8	−56	45	324		
SteerNr > HeadingNr	Cingulate gyrus	−9	−24	42	301	3.861	<0.05 (FDR)
	Superior parietal lobe	−9.6	−65	51	1782		
		−9	−72	47	274		
	Lateral occipitotemporal gyrus	−36	−67	−13	95		
SteerPv > HeadingPv	Cingulate sulcus	−8.9	−23	43	1157	3.379	<0.05 (FDR)
	Central sulcus	22	−28	55	734		
		−15	−30	61	454		
	Post central sulcus	26	−40	47	1121		
	Superior parietal lobe	13	−59	58	721		
		−24	−50	56	1491		
		−12	−59	52	628		
	Precuneus	9	−59	55	1383		
		−9	−48	49	998		
	Ventral intraparietal area	25	−66	33	1207		
	Middle occipital gyrus	36	−76	12	469		
	Medial occipitotemporal gyrus	31	−33	−17	912		
	Lingual gyrus	10	−31	−11	478		

P = 0.05 (FDR correction) unless otherwise stated.

#### Steering vs. heading

**Figure 2:**
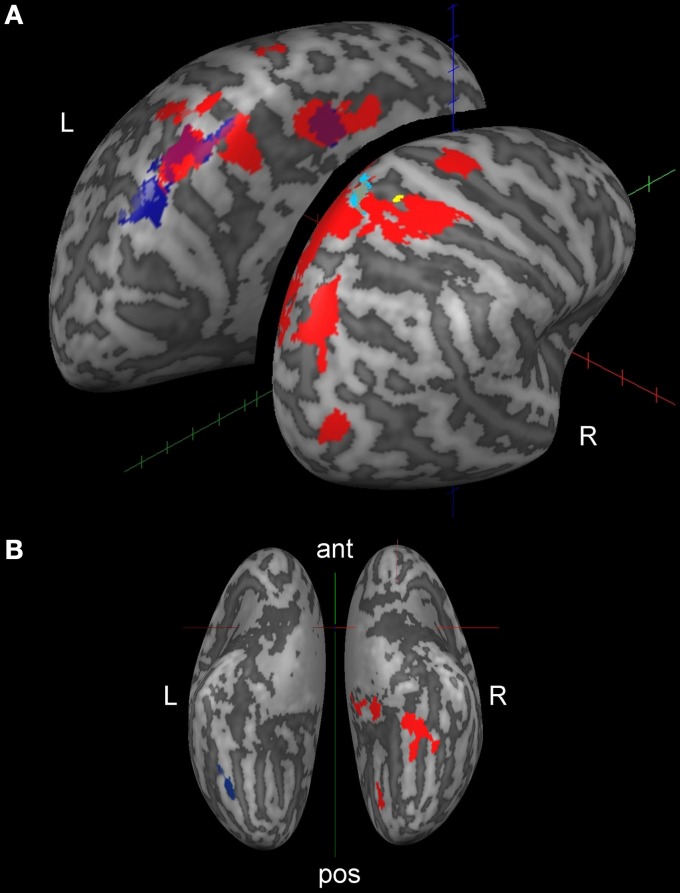
**Cortical activation displayed on inflated brain (lateral dorsal view, A; ventral view, B) for active steering trials.** The whole brain contrast SteerPv > HeadingPv is shown in red and the SteerNr > HeadingNr is shown in navy blue (no RH activation for this contrast). Both contrasts are shown at the FDR corrected = 0.05 level, exclusively masked with the PEF Localizer (Sacc > Fix, *p* < 0.05 unc.). Activations in yellow and light blue are discussed in Figure 4 and section “fMRI and behavioral performance correlations; steering”.

**Figure 3:**
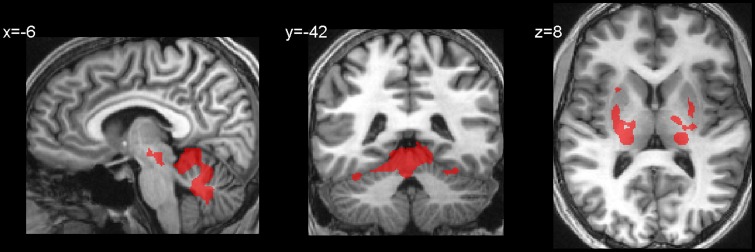
**Subcortical and hippocampal activations for the contrast SteerPv > HeadingPv (FDR, *p* < 0.05)**.

Table 2 and Figures 2A,B present activations for both the SteerNr > HeadingNr (*p* < 0.05, FDR, blue) and SteerPv > HeadingPv (*p* < 0.05, FDR, Red) contrasts, exclusively masked with the parietal eye field localizer maps (Sacc > Fix, *p* = 0.05 unc.). Both these contrasts reveal a BOLD response which is associated with actively steering, whilst being matched for low level visual features (i.e., presence of cones and optic flow) and motor features (joystick response). The contrast SteerNr > HeadingNr elicited activation in both cingulate gyrus and an extensive region of the SPL. The contrast SteerPv > HeadingPv activated both these cortical regions to a greater extent, with additional bilateral medial activation in the precuneus and lateral activation in the central sulci and postcentral gyri. Additional activation was also present in bilateral precentral sulci, right middle occipital gyrus (dorsal to MT+) and right IPS. Subcortically there were also activations coinciding with the pulvinar nucleus of the thalamus, ventral lateral thalamic nucleus and the putamen (Figure 3). The bilateral anterior cerebellum also displayed extensive activation (Figure 3).

#### fMRI and behavioral performance correlations; steering

**Figure 4:**
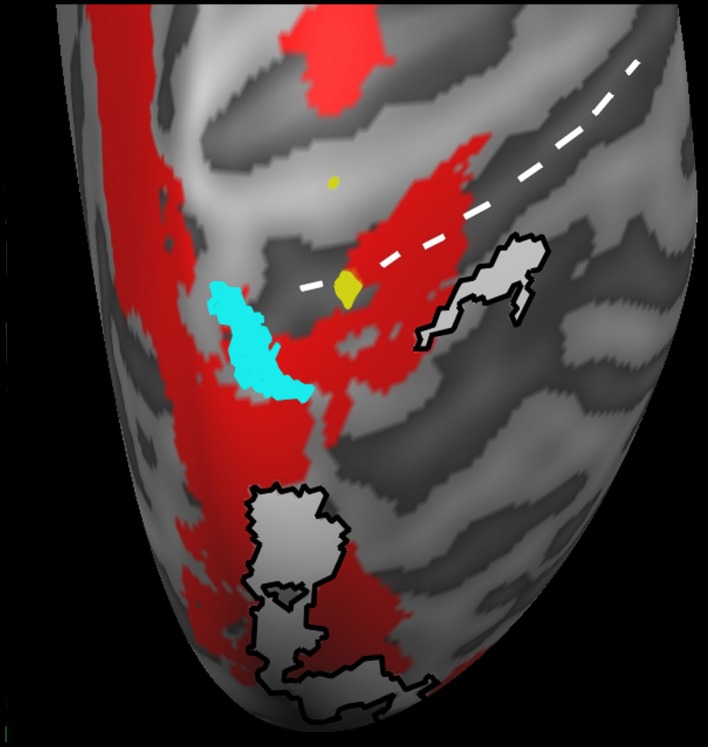
**A magnified dorsal view of the right hemisphere activation correlated with mean aJrk values during SteerPv trials (yellow; *p* < 0.001 unc.) and during SteerNr trials (turquoise; *p* < 0.001 unc.).** Activations are shown in relation to SteerPv > HeadingPv (red: FDR, *p* < 0.05) and activity during the Sacc > Fix contrast (White: *p* < 0.001 unc.) from the PEF localizer. The white dotted line indicates the postcentral sulcus.

An ANCOVA analysis was carried out in Brain Voyager in order to determine whether the differences in steering smoothness in individual participants contributed to differences in cortical activation. For this we explored the variation between the standardized aAcc and aJrk values and the activation (in terms of mean BOLD response) in either the SteerPv or SteerNr trials (*p* < 0.001 unc.). Figure 4 shows activation in the SPL, located in superior postcentral sulcus (Tal: *x* = 16, *y* = −50, *z* = 62) which was significantly predicted by the trajectory smoothness (aJrk) in the SteerNr trials (shown in cyan; ANCOVA coefficient = −0.408, *p* < 0.001) and marginally more lateral postcentral sulcus (Tal: *x* = 21, *y* = −42, 52) activation in SteerPv (shown in yellow; ANCOVA coefficient =−0.447, *p* < 0.001) trials. The negative correlation between the aJrk values and region of interest beta values suggests that participants recruiting this region were displaying the smoothest steering during the slalom task. No cortical activity correlated with aAcc scores.

#### fMRI and behavioral performance correlations; heading

In order to rule out the possibility that the aforementioned activations in SPL and postcentral sulcus merely reflected either individual variations in some mechanical aspects of steering with a joystick, or low level visual aspects of screen motion we examined aAcc and aJrk correlations during passive heading trials. The same regression analysis between aAcc and aJrk values and activation during HeadingNr and HeadingPv (vs. BL) did not reveal any cortical activity in associated with aAcc or aJrk scores. For heading trials lag values were regressed with activation in HeadingNr and HeadingPv (vs. BL) to determine cortical regions associated with maintaining timely joystick movements to on screen heading. Despite obvious individual differences in these scores (as highlighted by large *SD* values), no cortical activity was associated with these performance measures.

### Eyetracking results

**Figure 5:**
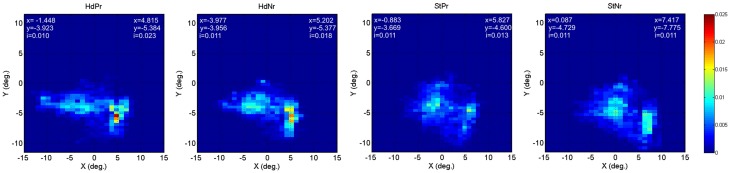
**Heat maps depicting relative eye gaze location (screen x°/y°) during HeadingPv, HeadingNr, SteerPv, and SteerNr trials.** Details of precise peak *x* and *y* positions for each visual hemifield are provided for each map [index (*i*) = peak decimal proportion value]. For the BL condition left hemifield values were *x* = −1.927, *y* = −4.371, *i* = 0.020; right hemi field values were *x* = 5.791, *y* = −4.868, *i* = 0.019.

Collated group eye tracking data are presented in two dimensional “heat maps” (Figure 5). Both Heading and Steering trials caused two distinct peaks in gaze position (lighter blue to red zones). Participants generally looked lower in the visual screen during SteerNr trials than during SteerPv trials, with both heading trials resulting in intermediate vertical gaze positions. These patterns were expected given that during SteerPv trials participants may be expected to attend to cones further in the distance (so higher on the display) as well as the current slalom cone. Because cones were irrelevant during heading trials, participants seemed more prone to gaze at a central point between the cones. Despite these apparent trends, an ANOVA revealed no significant differences in mean y gaze values across conditions [*F*_(4, 36)_ = 0.368, *p* = 0.830]. An equivalent regression analysis to that used for aAcc and aJrk was used for the mean *y* gaze values during SteerPv and SteerNr trails and this did not reveal any parietal cortical activity associated with the differences in gaze positions.

## Discussion

To our knowledge this is the first study to investigate how the human brain encodes and updates target locations and uses this visual information for the purpose of steering through an obstacle rich environment. This study also reveals that key cortical regions are differentially activated according to the smoothness of the steering trajectory.

### Path planning and smooth trajectories

In this study we were particularly interested in the behavioral and neural responses related to advance planning of the trajectory. When participants were presented with the whole slalom course at the start of the trial (SteerPv) they were able to plan ahead and so we would expect smooth trajectories, whereas when only the nearest slalom objects were visible (SteerNr) last minute responsive changes would have to be executed without future planning. It seems clear that near information should be more useful for immediate error correction whereas distant information about future waypoints allows heading to be anticipated (Billington et al., 2010) and smoother paths to be generated (Wilkie et al., 2008). In the present study there was a significant decrease in the rate of angular acceleration in the SteerPv compared to SteerNr. This suggests that, in general, participants were making fewer rapid adjustments to steering because they were able to use information regarding future obstacle location to execute more gradual adjustments.

### Neural correlates of steering

Actively driving through the slalom environment recruited cortical regions known to play a role in processing visual motion, spatial updating and somatosensory processing. The location of the cingulate gyrus activation for both SteerNr and SteerPv corresponds well with the region indentified as CSv (Wall and Smith, 2008). This region is thought to play a key role in detecting visual cues to egomotion (Wall and Smith, 2008; Cardin and Smith, 2010) and more recently it has been proposed that CSv has a role in integrating vestibular information for the purpose of canceling head motion cues during egomotion (Smith et al., 2011). This activation may therefore reflect the need to determine instantaneous heading whilst making steering judgments. We postulate that the relative increase in activation in CSv in the steering vs. heading conditions may be not only be due to small differences in optic flow components but may also be related to the fact that the motion is self-generated. This suggests that neurons in CSv are not being driven by visual input alone, but may receive online feedback from motor regions. Such an explanation seems plausible because the activation specific to the SteerPv task was anterior to the shared CSv activation, encroaching on the cingulate motor area, which has afferent connections with supplementary and primary motor cortices (Rizzolatti et al., 1998).

Activation common to both steering conditions was found in the central area of the precuneus, which is part of the SPL. The central precuneus region has previously been shown to exhibit strong connections to multimodal inferior parietal lobes regions and dorsolateral prefrontal cortex in humans. This activation may reflect a role in updating the visual location of objects in both conditions, which could be somewhat independent of intended actions (Wolbers et al., 2008). This suggests a role for the precuneus in forming representational maps of an object in egocentric space, and monitoring self-motion in space whilst steering. Predicting the location of a future obstacle once a more imminent obstacle has been circumvented could be important for a short period of time whilst steering in order to allow the timely and appropriate execution of a change in heading. In these circumstances spatial updating would be a predictive updating process rather than the continuous updating of the precise location of an unseen object.

### Neural correlates of path planning

When participants had access to future path information they were able to use this information in order to execute smoother steering trajectories, and we would expect such strategies to be reflected at the neural level. Indeed, the SteerPv condition elicited much more extensive activations in the SPL and IPS, as well as additional activations in the primary motor cortex (BA4), somatosensory cortex, occipital, occipitotemporal regions, and the cerebellum. The nature of activations in SPL is suggestive of the neural processes engaged during SteerPv compared to SteerNr. Activation during the SteerNr trials was more posterior, toward the parieto-occipital fissure. This region is thought to play a stronger role in visual processing, receiving inputs from areas of visual cortex, including MT, and having indirect connections through MT and MST to parietal regions such as VIP in the macaque (Colby et al., 1988). Activation specific to SteerPv was found at the anterior most part of the precuneus. This region shows connectivity to somatosensory regions of the SPL (Margulies et al., 2009). These central and anterior regions are thought to display similar functional architecture to PGm and PEc respectively in macaques (Margulies et al., 2009). Cells in PGm and PEc are modulated by self initiated hand movements in macaques (Ferraina et al., 1997) and a large proportion of cells in PEc respond to passive joint rotations, particularly in the upper limbs (Breveglieri et al., 2006). Thus, whilst SteerNr engaged predominantly visual and spatiotopic cortical regions, SteerPv engaged additional regions associated with self-initiated movement and motor planning.

Participants were allowed free eye movement during our experiment and, in general, SPL activation can often reflect the planning of eye movements (Kan et al., 2007) and attentional shifts (Corbetta et al., 1998). However, there was no significant difference in foci or spread of eye movements across conditions and the additional use of the Sacc localizer allowed us to confidently discount any activation which could have occurred due to additional eye movements in the steering conditions, particularly the SteerPv condition. Therefore, these activations reflect critical differences between SteerPv and SteerNr in relation to the heading conditions and suggest that integrating future targets and obstacles requires that the participants encode and update egocentric visual information in order to be able to make an appropriately executed motor response.

### Error detection

During steering trials participants were continually receiving feedback on the efficacy of motor commands on the basis of efferent information. The SteerPv trials required participants to continually adjust errors in heading in a manner that was not only appropriate to negotiate the immediate obstacle (as in SteerNr), but also optimal for traversing smoothly between the immediate obstacle and the next obstacle in the sequence based on their relative positions. Correction of ongoing movement using feedback loops is thought to recruit the IPS (Desmurget et al., 1999; Pisella et al., 2000). Pisella et al. (2000) required participants to make a smooth movement toward a target which was relocated after movement was initiated, and found that a patient with bilateral damage to these regions in the posterior parietal cortex (PPC) tended to point incorrectly to the initial target position before making a second corrective motion. Similar patterns of response have been found using TMS to temporarily disrupt the IPS (Desmurget et al., 1999). These studies suggest a role for corrective feedback mechanisms in the PPC and support our suggestion that corrective feedback loops assist efficient steering in our current task.

The cerebellum, thalamus, middle, and inferior frontal gyri (IFG) and IPS, and occipitotemporal cortex have also been implicated in playing a role in visual feedback control of ongoing motor movement (Inoue et al., 1998; Seidler et al., 2004; Ogawa et al., 2006). Aside from IFG, these areas were also activated during our SteerPv trials. Desmurget and Grafton (2000) implicate both the IPS and the cerebellum as key regions for making feedback strategies viable processes for fast effective motor responses. Crucial properties of the PPC, namely the ability to transform information from different modalities into a common coordinate system (Cohen and Andersen, 2002) and the ability to store representations of intended actions online (Fernandez-Ruiz et al., 2007) lend themselves to a similar role in the IPS (Desmurget and Grafton, 2000). Thus, actively steering recruits regions which may be involved in visuo-motor feedback, particularly when information regarding future goals and obstacles requires continual error correction in order to maintain smooth steering performance.

### Improved performance

A final question posed in this study was regarding whether individual differences in steering performance could be identified in specific cortical regions. We found two regions in the right superior postcentral sulcus in which greater activation was predicted by smoother steering performance. This somatosensory cortical area has a similar locality to that deemed to be putative human VIP (Sereno and Huang, 2006). Located in the superior part of the post central sulcus it is thought to contain topographically aligned maps of tactile and visual near space that are encoded in head centered coordinates (Cooke et al., 2003; Sereno and Huang, 2006). VIP has been found to play an important role in heading detection (Schaafsma and Duysens, 1996; Zhang et al., 2004; Wall and Smith, 2008; Zhang and Britten, 2011). Zhang and Britten (2010) point out that the ability of this region to both encode heading direction (which relies more on far field cues) and encode object location in the visual scene (which relies more on near field cues) is a perfect combination for the control of locomotion, and hence a plausible neural substrate for improved performance in the current study.

## Conclusions

This study has confirmed that a network of cortical areas play a role in effective locomotion by means of effective visuo-spatial encoding, visuo-motor encoding and integration and online corrective feedback mechanisms. In particular, the capability of neurons in regions of the SPL may allow for effective encoding of visual information regarding egocentric heading and obstacle location for the purpose of generating motor commands during locomotion. The importance of integrating visual and motor coordinates during locomotion is reinforced by our finding that participants who displayed smoother steering patterns showed greater activation in a region of the postcentral sulcus known to encode information from different sensory modalities. The IPS and cerebellum are engaged in order to estimate effector trajectories and compute error signals for correcting ongoing movements to support skilled motor actions. To spline a path through immediate and future waypoints multiple object goals have to be integrated and smoother steering is more appropriate. This paper implicates both dorsal steam processes and a parietal-cerebella network in not only supporting steering behaviors, but also contributing toward improved performance on an individual level.

### Conflict of interest statement

The authors declare that the research was conducted in the absence of any commercial or financial relationships that could be construed as a potential conflict of interest.
